# Benefit of Uracil–Tegafur Used as a Postoperative Adjuvant Chemotherapy for Stage IIA Colon Cancer

**DOI:** 10.3390/medicina59010010

**Published:** 2022-12-20

**Authors:** Po-Huang Chen, Hong-Jie Jhou, Chi-Hsiang Chung, Yi-Ying Wu, Tzu-Chuan Huang, Cho-Hao Lee, Wu-Chien Chien, Jia-Hong Chen

**Affiliations:** 1 Department of Internal Medicine, Tri-Service General Hospital, National Defense Medical Center, Taipei 11490, Taiwan; 2 Department of Neurology, Changhua Christian Hospital, Changhua 500, Taiwan; 3 School of Public Health, National Defense Medical Center, Taipei 11490, Taiwan; 4 Department of Medical Research, Tri-Service General Hospital, National Defense Medical Center, Taipei 11490, Taiwan; 5 Taiwanese Injury Prevention and Safety Promotion Association (TIPSPA), Taipei 114, Taiwan; 6 Division of Hematology and Oncology Medicine, Department of Internal Medicine, Tri-Service General Hospital, National Defense Medical Center, Taipei 11490, Taiwan; 7 Graduate Institute of Medical Sciences, National Defense Medical Center, Taipei 11490, Taiwan

**Keywords:** uracil–tegafur, colon cancer, chemotherapy, adjuvant therapy, stage IIA

## Abstract

*Background and Objectives*: Postoperative adjuvant therapy with uracil and tegafur (UFT) is often used for stage II colon cancer in Japan, but a limited number of studies have investigated the effects of UFT in these patients. *Materials and Methods*: We conducted a population-based cohort study in patients with resected stage II colon cancer comparing the outcomes after postoperative adjuvant chemotherapy with UFT with an observation-only group. The data were collected from the Taiwan National Health Insurance Research Database from 2000 to 2015. The outcomes of the study were disease-free survival (DFS) and overall survival (OS). The hazard ratios (HRs) were calculated using multivariate Cox proportional hazard regression models. Results: No differences in the DFS and OS were detected between the UFT (1137 patients) and observation (2779 patients) cohorts (DFS: adjusted HR 0.702; 95% confidence interval (CI) 0.489–1.024; *p* = 0.074) (OS: adjusted HR 0.894; 95% CI 0.542–1.186; *p* = 0.477). In the subgroup analyses of the different substages, UFT prolonged DFS in patients with stage IIA colon cancer (adjusted HR 0.652; 95% CI 0.352–0.951; *p* = 0.001) compared with DFS in the observation cohort, but no differences in the OS were detected (adjusted HR 0.734; 95% CI 0.475–1.093; *p* = 0.503). Conclusions: Our results show that DFS improved significantly in patients with stage IIA colon cancer receiving UFT as a postoperative adjuvant chemotherapy compared with DFS in the observation group.

## 1. Introduction

Colon cancer is third in incidence and cause of cancer deaths worldwide and has been increasing rapidly in recent decades [1]. Stage II cancers have no lymph node involvement or distal metastases, and radical surgical resection of the primary tumor is the standard treatment. The prognosis after resection is relatively favorable, with a 5-year disease-free survival (DFS) rate of approximately 68%–83% after surgery alone [2]. Adjuvant therapy may be considered after surgery for patients with a high risk of recurrence to eradicate micrometastatic disease [3]. The IDEA collaboration (International Duration Evaluation of Adjuvant) recently conducted the TOSCA (Three or Six Colon Adjuvant) trial to determine the optimal duration (3 months versus 6 months) of postoperative chemotherapy in patients with high-risk stage II or III radically resected colon cancer. The conclusion showed that it was still not debatable whether 3 months of oxaliplatin-based adjuvant treatment was as efficacious as 6 months; however, the difference in survival between the two treatment durations was small [4].

For years, postoperative adjuvant chemotherapy with fluorouracil (5-FU) has been a standard of care choice among patients with locally advanced colon cancer [5]. Tegafur-uracil (UFT) is an alternative postoperative adjuvant chemotherapy. UFT is an oral drug combination of tegafur, derived from 5-FU, and uracil in a molar ratio of 1:4. UFT acts as a competitive inhibitor of dihydropyrimidine dehydrogenase (DPD) [6]. Because of UFT’s tolerability and safety in an outpatient setting, it is the most commonly prescribed adjuvant chemotherapeutic for colon cancer in Taiwan [7]. A nationwide cohort study and meta-analysis demonstrated the similar effects of UFT and intravenous 5-FU on DFS and overall survival (OS), but UFT has a lower incidence of adverse events when used as a postoperative adjuvant chemotherapy in patients with locally advanced colon cancer [8].

Adjuvant chemotherapy represents a dilemma for clinical oncologists when treating patients with stage II colon cancer receiving surgical resection. In previous studies, the survival benefits of adjuvant chemotherapy were still inconclusive in unselected stage II patients. The adjuvant therapy benefits may be limited in patients with average-risk stage II cancer and a relatively good prognosis. According to the 2021 American Society of Clinical Oncology guidelines, adjuvant chemotherapy is not recommended for patients with a low risk of recurrence [9]. Thus, high-risk features should be identified to determine which subgroups might benefit from adjuvant chemotherapy. The current guidelines suggest that patients with one or more high-risk features should receive adjuvant chemotherapy, including pT4, bowel obstruction, or tumor perforation; fewer than 12 lymph nodes harvested; vascular, lymphatic, or perineural invasion; and a poorly differentiated histology [2]. However, the relative prognostic weight of these features is not considered.

Although postoperative adjuvant therapy with UFT is often used for stage II colon cancer in Japan, a limited number of studies have investigated the effects of UFT in these patients. Thus, clarifying whether postoperative adjuvant treatment with UFT is beneficial in stage II colon cancer patients at different substages compared with observation alone in a larger population, and a real-world setting is needed. This study, using a population-based database, aimed to examine the survival benefit of oral UFT compared with observation only for postoperative stage II colon cancer patients analyzed at different substages.

## 2. Materials and Methods

### 2.1. Data Source

The data were gathered from databases provided by the Health and Welfare Data Science Center, including the Taiwan Cancer Registry (TCR) Database 2000–2015 [10] and the National Health Insurance Research Database (NHIRD) 2000–2015.

The TCR is organized and funded by the Ministry of Health and Welfare and is managed by the Taiwan Public Health Association. All hospitals in Taiwan with at least 50 beds are required to report all newly diagnosed and confirmed malignancies to the registry. Detailed information on diagnosis, treatments, and outcomes is collected from 80 hospitals, covering more than 90% of all cancer cases diagnosed annually in Taiwan. Diagnoses are coded according to the International Classification of Diseases for Oncology, 3rd Edition, format [11].

A nationwide population-based study was conducted using data from 2000 to 2015 obtained from the Longitudinal Health Insurance Database (LHID) of Taiwan [12]. Two million beneficiaries from the NHIRD registry were randomly sampled. The LHID includes the following claims data: sociodemographic information, medical visits, emergency care, hospitalization, surgical procedure, medication, and other medical services. Diseases are diagnosed according to the International Classification of Diseases, Ninth Revision, Clinical Modification (ICD-9-CM) codes. The Registry for Catastrophic Illness Patient Database (RCIPD) includes data from insured residents with severe diseases, such as malignancies, as defined by the NHI program [13]. 

This study was approved by the Institutional Review Board of the Tri-Service General Hospital, Taipei, Taiwan (TSGHIRB No. B-110-12). Because the data from the NHI were de-identified, the signed informed consent of the included patients was waived. 

### 2.2. Study Population and Definition of Statin Exposure

Patients newly diagnosed with colon cancer (ICD-9 Code: 153–154.1) from 1 January 2000 to 31 December 2015, were identified from the NHIRD database. The malignancy diagnosis was confirmed using the RCIPD data. Patients with stage II colon cancer who received operative therapy within 6 months after the diagnosis (ICD-9-CM Procedure Code: OP 45.21, OP 45.71–45.76, OP 45.79, OP 45.8, and OP 48.4–48.6) were identified from the TCR database, and their data were retrieved. The TCR uses the American Joint Committee on Cancer staging system, 7th Edition, to record the stages of all cancer patients. We excluded patients that received other target or chemotherapies, including bevacizumab, cetuximab, capecitabine, irinotecan, oxaliplatin, or 5-FU; diagnosed cancer before the index date using ICD-9-CM140–239; diagnosed secondary malignancy (ICD-9-CM: 196–198.9); or benign colon neoplasm (ICD-9-CM: 211.3 and 211.4). The UFT cohort comprised patients who were prescribed UFT, and the observation (without treatment) cohort comprised patients who did not receive any postoperative chemotherapy.

### 2.3. Outcome and Comorbidities Measurement

The outcomes of interest were the DFS and the OS. DFS was defined as the time interval from the first day postoperation to tumor recurrence or death. OS was defined as the time interval from the first day postoperation to death. We also extracted the covariates of the patients, including age, sex, stage, and underlying diseases. The Charlson Comorbidity Index (CCI) categorizes the comorbidities of patients based on the ICD codes [14]. The CCI Revised (CCI_R) was calculated by removing the variables mentioned and accounted for in the baseline comorbidities. The socioeconomic status of the study participants was approximated using insurance premiums (i.e., income level), level of care (stratified by the levels of hospital, including central, regional, or local hospitals determined by the Taiwanese government), and urbanization levels [15]. Additional analyses were conducted to ascertain the impact of adjuvant chemotherapy on DFS and OS in patients at different substages (stage IIA/IIB/IIC) in the UFT and observation groups.

### 2.4. Statistical Analysis

The categorical variables are expressed using numbers (i.e., percentages), and the continuous variables are expressed as the mean ± standard deviations (SDs). The chi-square test or Fisher’s exact test was used to compare the categorical variables, whereas *t*-tests were used to compare the mean difference for continuous variables among the UFT and observation groups. Univariate and multivariate Cox regression analyses were employed to evaluate the crude and adjusted hazard ratios (HRs) for the influence (odds) of the analyzed variables on DFS and OS; the observation group was used as a reference. We adjusted the multivariate Cox regression model using all of the characteristics, including age; sex; insurance premium; level of care; urbanization; comorbidities, including hypertension, diabetes mellitus (DM), chronic obstructive pulmonary disease (COPD), chronic kidney disease (CKD), ischemic heart disease (IHD), congestive heart disease (CHD), and stroke; and a CCI_R. Kaplan–Meier analysis and log-rank tests of DFS and OS based on the stage of colon cancer were performed. The two-sided *p*-values of the log-rank test less than 0.05 were considered statistically significant. All statistical analyses were performed using IBM SPSS Statistics for Windows version 22.0 (IBM Corp., Armonk, NY, USA).

## 3. Results

**Figure 1:**
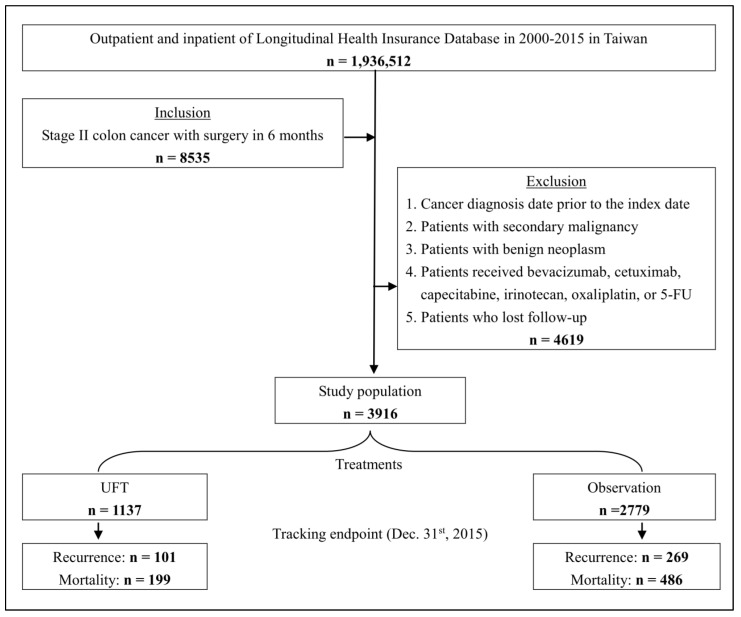
A flow chart of the recruitment of the subjects.

A total of 3916 surgical patients with stage II colon cancer were observed in this study, including 1137 patients in the UFT group and 2779 patients in the observation group. Figure 1 shows a flow chart of the recruitment of subjects from the NHIRD.

### 3.1. Patient Characteristics

Table 1 shows the characteristics and baseline comorbidity status of the UFT (n = 1137) and observation (n = 2779) cohorts. The percentages of males in the UFT and observation cohorts were 59.89% and 58.94%, respectively. The mean ± SD ages for the UFT and observation cohorts were 63.40 ± 10.25 and 65.12 ± 11.12 years, respectively. The mean follow-up periods were 7.20 ± 6.84 and 7.22 ± 6.87 years in the UFT and observation cohorts, respectively. No significant differences in sex; age; insured premium; urbanization; or comorbidities, including hypertension, DM, COPD, CKD, IHD, CHD, and stroke; or CCI_R index were detected (the social–economic data are shown in Supplementary Materials Table S1; the follow-up period data are shown in Supplementary Materials Table S2A,B; the ICD-9-CM, NHI code, and definition are shown in Supplementary Material Table S3).

### 3.2. Disease-Free Survival

**Figure 2:**
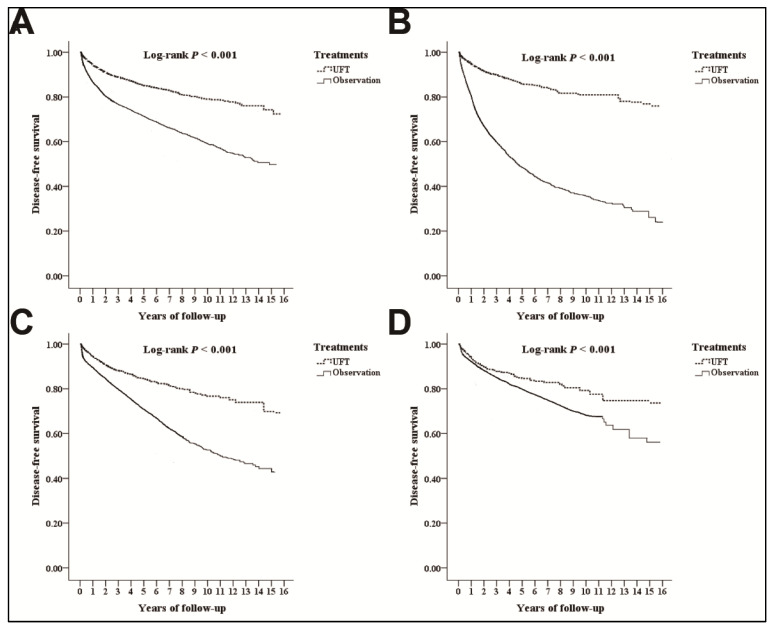
Kaplan–Meier plots for cumulative risk in disease-free survival: overall populations (**A**); Stage IIA (**B**); Stage IIB (**C**); Stage IIC (**D**).

The Kaplan–Meier plots with log-rank tests revealed significant differences in DFS between the UFT and observation cohorts (log-rank test: *p* < 0.001; Figure 2). According to the multivariate Cox regression model, DFS did not differ significantly between the UFT and observation groups (UFT vs. observation; adjusted HR 0.702; 95% CI 0.489–1.024; *p* = 0.074; Table 2). Male sex, having comorbidities (i.e., HTN, DM, CKD, IHD, CHD, and stroke), and the influence of the CCI_R score were significant factors with shorter DFS.

### 3.3. Overall Survival

**Figure 3:**
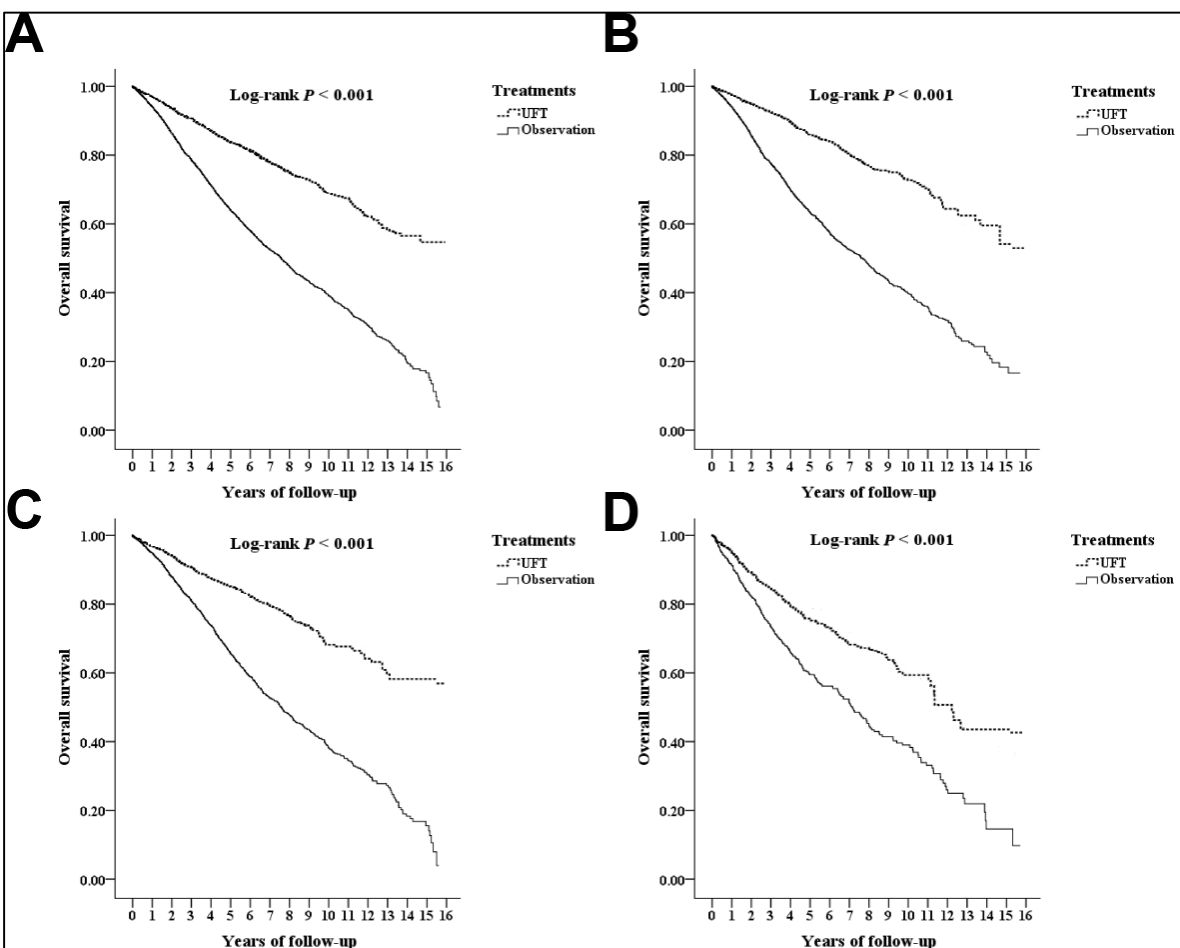
Kaplan–Meier plots for the cumulative risk in overall survival: overall populations (**A**); Stage IIA (**B**); Stage IIB (**C**); Stage IIC (**D**).

The Kaplan–Meier plots with log-rank tests revealed significant differences in the OS between the UFT and observation cohorts (log-rank test: *p* < 0.001; Figure 3). 

The multivariate Cox regression model indicated that the OS did not differ significantly between the UFT and observation groups (adjusted HR 0.894; 95% CI 0.542–1.186; *p* = 0.477; Table 2). Male sex, older age, having comorbidities (i.e., HTN, DM, CKD, IHD, CHD, and stroke), and the influence of the CCI_R score were significant factors with shorter OS.

### 3.4. Analyses for the Different Substages

Among the patients with stage IIA colon cancer, the Kaplan–Meier plots with log-rank tests revealed significant differences in the DFS (log-rank test: *p* < 0.001; Figure 2) and OS (log-rank test: *p* < 0.001; Figure 3) between the UFT and observation cohorts. The multivariate Cox regression model indicated that the DFS increased significantly in patients with stage IIA colon cancer receiving postoperative UFT adjuvant chemotherapy compared with the DFS in the observation group (adjusted HR 0.652; 95% CI 0.352–0.951; *p* = 0.001; Table 3); however, no differences in the OS were detected (adjusted HR 0.734; 95% CI 0.475–1.093; *p* = 0.503; Table 3)

In patients with stages IIB and IIC colon cancer, the Kaplan–Meier plots with log-rank tests revealed significant differences in the DFS (both *p* < 0.001; Figure 2) and OS (both *p* < 0.001; Figure 3) between the UFT and observation cohorts. According to the multivariate Cox regression model in the patients with stages IIB and IIC colon cancer, no difference in the DFS and OS between the UFT and observation groups were detected (DFS: stage IIB, adjusted HR 0.713, 95% CI 0.492–1.029, *p* = 0.079; DFS: stage IIC, adjusted HR 0.804, 95% CI 0.575–1.125, *p* = 0.184; Table 2) (OS: stage IIB, adjusted HR 0.877; 95% CI 0.531–1.153, *p* = 0.483; OS: stage IIC, adjusted HR 0.904, 95% CI 0.638–1.256, *p* = 0.425; Table 3).

## 4. Discussion

This nationwide, large-scale, retrospective cohort study compared the effectiveness of postoperative adjuvant chemotherapy with UFT to observation only in stage II colon cancer patients. In the 15-year follow-up cohorts, UFT showed no difference with observation only in DFS and OS. However, in the subgroup analysis of stage IIA, the patients who received UFT as a postoperative adjuvant chemotherapy had significantly prolonged DFS compared with observation alone.

### 4.1. UFT Effectiveness

Currently, 5-FU (5-FU/LV, capecitabine, UFT, and S-1) and oxaliplatin are the main drugs used as postoperative adjuvant chemotherapy for colon cancer. The usual dosage of intravenous 5-FU is a weekly 24 hour infusion of a maximal tolerable dose of 5-FU (2600 mg/m^2^) and LV (500 mg/m^2^) for 6 months [16]. Two oral UFT capsules, containing tegafur 100 mg and uracil 224 mg, are administered twice per day (400 mg of tegafur per day) in Taiwan [17]. A 5-day treatment plus a 2-day rest regimen of UFT for 12 months was beneficial in the NSAS-CC study [18]. In contrast, the NSABP C-06 study showed that oral UFT had DFS and OS similar to those of intravenous 5-FU/LV. However, patients treated with UFT had a better quality of life than those treated with 5-FU/LV [19]. 

### 4.2. Survival Paradox in Patients with Localized Advanced Colon Cancer

A survival paradox was noted between stage IIB/C (T4N0) and stage IIIA (T1-2N1 and T1N2a) colon cancer in previous studies [20,21,22,23]. Li et al. [24] found that the colon-cancer-specific survival (CCSS) rate of the stage IIIA colon cancer patients were significantly higher than that of the stage IIB and IIC colon cancer patients (5-year CCSS rates for stage IIB vs. stage IIC vs. stage IIIA: 74.2% vs. 72.5% vs. 91.9%). Furthermore, Mo et al. analyzed data from the US Surveillance, Epidemiology, and End Results (SEER) database, and showed that patients with stage IIA rectal cancer had worse survival than patients with stage IIIA disease [25]. The inferior survival in stage II compared with stage IIIA may be due to the lower use of systemic chemotherapy in stage II colon cancer patients. Thus, we should improve the survival of stage II colon cancer patients, especially in stage IIA. The 5-year DFS improved in patients with low-risk IIA colon cancer after receiving adjuvant chemotherapy with UFT more than 12 months after surgery in a retrospective cohort study [26], which is consistent with our finding.

### 4.3. Survival Risk Factors in Patients with Localized Advanced Colon Cancer

Over the past decades, subgroup analyses from large adjuvant trials have investigated the impacts of risk factors in prognosis [27]. The crucial prognostic factors for disease progression included T stage as tumor size, lymph node status, pathological grading, and microsatellite status.

The T stage is highly associated with tumor size. In a study conducted by Mo et al. [25], the mean tumor size of stage IIA rectal cancer was larger than the tumor size of stage IIIA rectal cancer in both the SEER and FUSCC cohorts. Therefore, en bloc resection of T1/T2 tumors may be much easier to achieve than the resection of T3/T4 tumors, making surgical negative margins more difficult to achieve for a high T level stage IIA colon cancer than a low T level IIIA disease. The larger tumor size in stage IIA colon cancer can increase the surgical margin positivity, thus escalating the recurrence rate of colon cancer and jeopardizing the prognosis of colon cancer patients.

Lymph node status is a crucial prognostic factor in colorectal cancer to determine postoperative managements and follow-up plans [28,29]. Stage III is distinguished from stage II colorectal cancer by the presence of lymph node metastases. According to the recommendation by the American Joint Committee on Cancer and the College of American Pathologists, at least 12 lymph nodes should be examined to adequately stage colorectal cancer patients [30,31]. Examining an adequate number of lymph nodes has been regarded as a key quality measure for colon cancer care in the United States since 2006 [32]. 

A high grade, indicating poorly differentiated disease, is associated with poor prognosis [33]. In a cohort of 3302 stage II and stage III colon cancer patients, Gill et al. observed lower 5-year DFS and OS in high-grade disease. In addition, high-grade disease was related to a loss of 8%–9% in 5-year DFS in T3N0 and T4N0 tumors compared with low-grade disease (65% vs. 73% and 51% vs. 60%, respectively) [34].

Two groups of colorectal cancers can be distinguished based on the state of mismatch repair: MSI-high (MSI-H, deficiency of the mismatch repair) and MSI-low (proficiency of the mismatch repair, pMMR). Adjuvant chemotherapy is not suggested for MSI-H stage II patients without high-risk features; therefore, observation is considered a reasonable treatment option [35].

### 4.4. Limitations

Several limitations should be considered when interpreting the results of this study. First, the NHIRD had insufficiently detailed clinical data, including the severity of lymph node involvement, pathologic grade, microsatellite instability status, the reasons for each patient’s treatment plan, or the quality of surgery. Second, patients with cancer were defined using claims data and diagnostic codes. The diagnostic accuracy remained unclear, and disease misclassification might cause false associations [36]. Third, the National Quality Forum has listed the assessment of at least 12 lymph nodes among the key quality measures for colon cancer care in the United States since 2006. However, data for this study were from 2000 to 2015; hence, the number of lymph nodes examined may be insufficient. Finally, potential confounders may have occurred that could bias the results. There remains a need to perform further analysis using the clinical data from individual participants and controlling for potential confounders [37].

To the best of our knowledge, our study is the first real-world study to examine the effectiveness of UFT in stage II colon cancer and its substages and a large-scale study to strengthen the statistical power [38]. A stratified analysis was conducted using demographic characteristics, including age, sex, socioeconomic status, and comorbidities, to exam the clinical heterogenicity. 

## 5. Conclusions

Our results show that the DFS improved significantly in patients with stage IIA colon cancer receiving UFT as a postoperative adjuvant chemotherapy compared with the DFS in the observation group.

## Figures and Tables

**Table 1:** Characteristics of study in the baseline.

Treatment Variables		UFT		Observation		*p-*Value
		n	%	n	%	
Total		1137	29.03	2779	70.97	
Gender						0.582
Male		681	59.89	1638	58.94	
Female		456	40.11	1141	41.06	
Age (years ± SD)		63.40 ± 10.25		65.12 ± 11.12		<0.001
HTN	With	330	29.02	781	28.10	0.562
	Without	807	70.98	1998	71.90	
DM	With	201	17.68	455	16.37	0.321
	Without	936	82.32	2324	83.63	
COPD	With	55	4.84	108	3.89	0.186
	Without	1082	95.16	2671	96.11	
CKD	With	17	1.50	36	1.30	0.648
	Without	1120	98.50	2743	98.70	
IHD	With	60	5.28	145	5.22	0.937
	Without	1077	94.72	2634	94.78	
CHD	With	24	2.11	54	1.94	0.733
	Without	1113	97.89	2725	98.06	
Stroke	With	33	2.90	80	2.88	0.968
	Without	1104	97.10	2699	97.12	
CCI_R		1.03 ± 0.19		1.03 ± 0.15		0.998

*p*-Value: categorical variables: chi-squared/Fisher’s exact test; continuous variables: *t*-test. UFT, uracil–tegafur; HTN, hypertension; DM, diabetes mellitus; COPD, chronic obstructive pulmonary disease; CKD, chronic kidney disease; IHD, ischemic heart disease; CHD, congestive heart disease; CCI_R, Charlson comorbidity index revised.

**Table 2:** Cox regression analysis of disease-free survival and overall survival.

Prognosis	Disease-Free Survival (DFS)				Overall Survival (OS)			
Variables	Crude HR (95% CI)	*p*	Adjusted HR (95% CI)	*p*	Crude HR (95% CI)	*p*	Adjusted HR (95% CI)	*p*
**Treatments**								
UFT vs. observation	0.613 (0.377–0.806)	<0.001	0.702 (0.489–1.024)	0.074	0.785 (0.426–0.894)	<0.001	0.894 (0.542–1.186)	0.477
**Gender**								
Male vs. female	1.365 (1.124–1.503)	<0.001	1.265 (1.106 –1.482)	<0.001	1.489 (1.303 –1.677)	<0.001	1.420 (1.298–1.583)	<0.001
**Age Groups (Years)**								
<30	Reference							
30–39	1.124 (0.822–1.825)	0.182	1.024 (0.724–1.781)	0.389	2.561 (1.786 –4.486)	<0.001	2.008 (1.025–3.349)	0.035
40–49	1.304 (0.913–1.911)	0.094	1.203 (0.902–1.924)	0.172	3.789 (2.229–5.702)	<0.001	2.186 (1.097–3.570)	0.001
50–59	1.386 (0.972–1.934)	0.067	1.186 (0.851–1.876)	0.234	5.978 (3.224–9.972)	<0.001	4.299 (2.004–8.301)	<0.001
≧60	1.402 (1.020–2.020)	0.030	1.354 (0.989–1.986)	0.069	7.124 (4.809–13.312)	<0.001	5.038 (2.897–9.896)	<0.001
**HTN**	1.678 (1.307–1.882)	<0.001	1.562 (1.265–1.782)	<0.001	1.863 (1.511–2.104)	<0.001	1.782 (1.428–2.006)	<0.001
**DM**	1.831 (1.367–2.010)	<0.001	1.762 (1.303–1.977)	<0.001	2.030 (1.724–2.308)	<0.001	1.975 (1.629–2.210)	<0.001
**COPD**	1.382 (0.986–1.769)	0.072	1.283 (0.865–1.677)	0.277	1.397 (1.002–1.784)	0.049	1.270 (0.852–1.624)	0.289
**CKD**	1.482 (1.153–1.780)	<0.001	1.293 (1.021–1.445)	0.029	2.156 (1.503–2.970)	<0.001	2.011 (1.452–2.897)	<0.001
**IHD**	1.686 (1.112–1.897)	<0.001	1.553 (1.086–1.795)	0.002	1.918 (1.628–2.774)	<0.001	1.897 (1.583–2.610)	<0.001
**CHD**	1.735 (1.442–1.975)	<0.001	1.652 (1.352–1.896)	<0.001	1.993 (1.586–2.601)	<0.001	1.824 (1.550–2.533)	<0.001
**Stroke**	1.808 (1.553–2.030)	<0.001	1.771 (1.448–1.909)	<0.001	2.030 (1.724–2.789)	<0.001	1.902 (1.652–2.672)	<0.001
**CCI_R**	1.304 (1.205–1.488)	<0.001	1.246 (1.112–1.304)	<0.001	1.372 (1.289–1.483)	<0.001	1.297 (1.158–1.372)	<0.001

HR, hazard ratio; CI, confidence interval; adjusted HR, adjusted variables listed in the table.

**Table 3:** Factors of prognosis stratified by cancer stage.

UFT vs. Observation		Disease-Free Survival (DFS)			
Stage	Patients	Adjusted HR	95% CI	95% CI	*p*
Overall	3916	0.702	0.489	1.024	0.074
Stage IIA	2326	0.652	0.352	0.951	0.001
Stage IIB	819	0.713	0.492	1.029	0.079
Stage IIC	871	0.804	0.575	1.125	0.184
		**Overall Survival (OS)**			
**Stage**	**Patients**	**Adjusted HR**	**95% CI**	**95% CI**	***p***
Overall	3916	0.894	0.542	1.186	0.477
Stage IIA	2326	0.734	0.475	1.093	0.503
Stage IIB	819	0.877	0.531	1.153	0.483
Stage IIC	871	0.904	0.638	1.256	0.425

Adjusted HR, adjusted hazard ratio (adjusted for the variables listed in Table 2); CI, confidence interval.

## Data Availability

Data are available from the National Health Insurance Research Database (NHIRD) published by Taiwan National Health Insurance (NHI) Bureau. Due to legal restrictions imposed by the government of Taiwan in relation to the “Personal Information Protection Act”, data cannot be made publicly available. Requests for data can be sent as a formal proposal to the NHIRD (http://nhird.nhri.org.tw).

## Supplementary Materials

The following are available online at https://www.mdpi.com/article/10.3390/medicina59010010/s1.
