# Supplementary material for: Benefit of Uracil–Tegafur Used as a Postoperative Adjuvant Chemotherapy for Stage IIA Colon Cancer
Source: Medicina (Kaunas). 2022 Dec 20;59(1):10. doi: 10.3390/medicina59010010 (PMC9864689; doi:10.3390/medicina59010010)
Supplement: Supplementary file 1 [file medicina-59-00010-s001.zip › medicina-2054139-supplementary.pdf]

Supplementary Appendix

Contents

Supplementary Table S1. Characteristics of study in the baseline - Social economic data

Supplementary Table S2. Years of follow-up and Years to events

Supplementary Table S3. ICD-9-CM, NHI code and definition

Supplementary Table S1. Characteristics of study in the baseline - Social economic data

**Treatment Variables**	**Total**		**UFT**		**5-FU**		***p-value***
	**n**	**%**	**n**	**%**	**n**	**%**	
Total	3,916		1,137	29.03	2,779	70.97	
Insured premium (NT$)							0.059
<18,000	3,818	97.50	1,098	96.57	2,720	97.88	
18,000-34,999	60	1.53	24	2.11	36	1.30	
≧35,000	38	0.97	15	1.32	23	0.83	
Urbanization level							0.905
1 (The highest)	1,525	38.94	444	39.05	1,081	38.90	
2	1,874	47.85	543	47.76	1,331	47.89	
3	206	5.26	56	4.93	150	5.40	
4 (The lowest)	311	7.94	94	8.27	217	7.81	
Level of care							0.044
Hospital center	2,164	55.26	663	58.31	1,501	54.01	
Regional hospital	1,501	38.33	403	35.44	1,098	39.51	
Local hospital	251	6.41	71	6.24	180	6.48	

p-value**: categorical variables: chi-squared/Fisher's exact test; continuous variables: t-test**

Supplementary Table S2. Years of follow-up and Years to events

Supplementary Table S2A. Years of follow-up				
**Treatments**	**Min**	**Median**	**Max**	**Mean ± SD**
UFT	0.01	4.82	15.92	7.20 ± 6.84
Observation	0.01	4.83	15.92	7.22 ± 6.87
Overall	0.01	4.85	15.92	7.23 ± 6.88

Supplementary Table S2B. Years to events								
**Events**	**DFS**				**OS**			
**Treatments**	**Min**	**Median**	**Max**	**Mean ± SD**	**Min**	**Median**	**Max**	**Mean ± SD**
UFT	0.08	1.95	15.74	2.58 ± 2.49	0.02	3.28	15.84	4.39 ± 3.52
Observation	0.08	1.84	15.83	2.50 ± 2.33	0.02	3.20	15.85	4.28 ± 3.43
Overall	0.08	1.92	15.83	2.55 ± 2.41	0.02	3.22	15.85	4.33 ± 3.47

Supplementary Table S3. ICD-9-CM, NHI code and definition

	**Abbreviation**	**ICD-9-CM / NHI code / definition**
**Stduy population**		
Colon cancer		153-154.1
Surgery		OP45.21, OP45.71-OP45.76, OP45.79, OP45.8, OP48.4-OP48.6
**Excluding:**		
Other cancers		140-239, excluding colon cancer
Secondary malignancy		196-198.9
Benign neoplasm of the colon		211.3-211.4
Other medicine		Bevacizumab, Cetuximab, Capecitabine, Irinotecan, Oxaliplatin, Fluorouracil
**Treatments**		Within 3-months after colorectal cancer surgery
Oral	UFT	Uracil and tegafur
**Events:** Prognosis		
Recurrence		196.1-198.89
Mortality		
**Comorbidities**		
Hypertension	HTN	401-405
Dabetes mellitus	DM	250
Chronic obstructive pulmonary disease	COPD	490-496
Chronic kidney disease	CKD	585
Ischemic heart disease	IHD	410-414
Congestive heart disease	CHD	428-429

Stroke		430-438
**Charlson comorbidity index revised**	CCI_R	CCI removed cancer, HTN, DM, COPD, CKD, IHD, CHD, and stroke
